# Chronic Sleep Disruption Alters Gut Microbiota, Induces Systemic and Adipose Tissue Inflammation and Insulin Resistance in Mice

**DOI:** 10.1038/srep35405

**Published:** 2016-10-14

**Authors:** Valeriy A. Poroyko, Alba Carreras, Abdelnaby Khalyfa, Ahamed A. Khalyfa, Vanessa Leone, Eduard Peris, Isaac Almendros, Alex Gileles-Hillel, Zhuanhong Qiao, Nathaniel Hubert, Ramon Farré, Eugene B. Chang, David Gozal

**Affiliations:** 1 Section of Pediatric Sleep Medicine, Department of Pediatrics, Pritzker School of Medicine, Biological Sciences Division, The University of Chicago, Chicago, IL 60637, USA; 2 Department of Medical Oncology, City of Hope, Duarte, CA, 91010, USA; 3 Department of Medicine, The University of Chicago, Chicago, IL 60637, USA; 4 Centro de Investigación Biomédica en Red de Enfermedades Respiratorias, CIBER, Madrid, Spain; 5 Institut Investigacions Biomediques August Pi Sunyer, Barcelona, Spain; 6 Unitat de Biofísica i Bioenginyeria, Facultat de Medicina, Universitat de Barcelona-IDIBAPS, Barcelona, Spain

## Abstract

Chronic sleep fragmentation (SF) commonly occurs in human populations, and although it does not involve circadian shifts or sleep deprivation, it markedly alters feeding behaviors ultimately promoting obesity and insulin resistance. These symptoms are known to be related to the host gut microbiota. Mice were exposed to SF for 4 weeks and then allowed to recover for 2 weeks. Taxonomic profiles of fecal microbiota were obtained prospectively, and conventionalization experiments were performed in germ-free mice. Adipose tissue insulin sensitivity and inflammation, as well as circulating measures of inflammation, were assayed. Effect of fecal water on colonic epithelial permeability was also examined. Chronic SF-induced increased food intake and reversible gut microbiota changes characterized by the preferential growth of highly fermentative members of *Lachnospiraceae* and *Ruminococcaceae* and a decrease of *Lactobacillaceae* families. These lead to systemic and visceral white adipose tissue inflammation in addition to altered insulin sensitivity in mice, most likely via enhanced colonic epithelium barrier disruption. Conventionalization of germ-free mice with SF-derived microbiota confirmed these findings. Thus, SF-induced metabolic alterations may be mediated, in part, by concurrent changes in gut microbiota, thereby opening the way for gut microbiome-targeted therapeutics aimed at reducing the major end-organ morbidities of chronic SF.

Fragmented sleep (SF) occurs frequently in many highly prevalent disorders, e.g., obstructive sleep apnea (OSA), and leads to excessive daytime sleepiness as well as to cognitive, mood, and neurobehavioral deficits^1^. SF also imposes adverse metabolic consequences, such as increased appetite and food intake that ultimately promote the emergence of obesity and metabolic dysfunction, the latter further exacerbating sleep disruption^2^,^3^. Indeed, even before the SF-induced increase in somatic weight gain occur, evidence of altered insulin sensitivity is apparent in adipose tissues and has been ascribed to increased SF-induced oxidative stress and inflammatory processes^4^. However, the mechanisms underlying the emergence of visceral white adipose tissue (VWAT) inflammation in the context of disrupted sleep are unclear and could potentially involve alterations in the gut microbiome.

The gut microbiome^5^ has been identified as playing a significant role in the phenotypic characteristics of host, immunity, metabolism^6^, and circadian clock function^7^. Furthermore, complex disorders previously attributed to lifestyle^8^,^9^,^10^ are now claimed as microbiota-related. In a now large and ever increasing number of human and animal studies changes in gut microbial communities have been mechanistically linked with adiposity^8^,^11^. The cumulative evidence from these studies has led to a generalized consensus that gut microbiota mediates an energy intake^12^, and play a critical role in the development of the “metabolic phenotype”^11^. We have recently demonstrated that exposures to intermittent hypoxia mimicking OSA lead to profound alterations in gut microbiota^13^. Therefore, we hypothesized that the disrupted patterns of sleep that characterize OSA, as well as many other sleep disorders, may be linked to obesity and metabolic phenotypes via changes in gut microbial communities. Here we explored the effects of SF on gut microbiome and the downstream metabolic implications of such effects in visceral adipose tissues.

## Results

### SF for 4 weeks increases food intake, insulin resistance, and VWAT mass and inflammation without body weight changes

**Figure 1:**
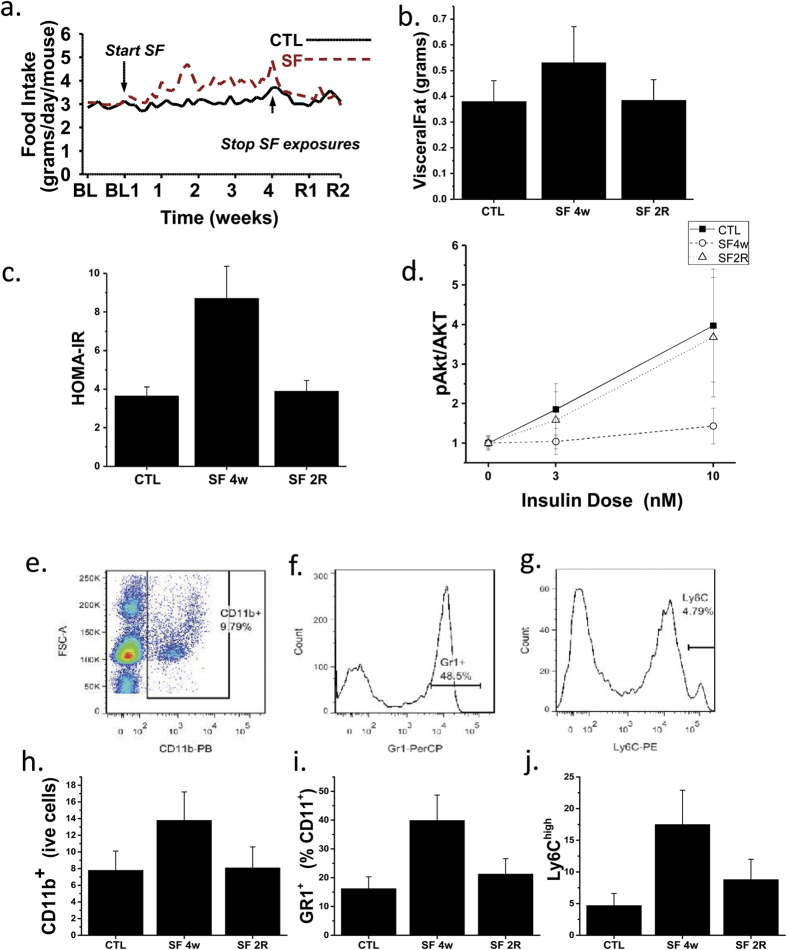
SF exposure increases food intake over time, which returns to baseline upon discontinuation of SF (**a**), along with increased VWAT mass (**b**), systemic (**c**) and VWAT insulin resistance (**d**). Representative FACS plots showing the gating strategies used for macrophages (**e**), neutrophils (**f**), and M1 macrophages (**g**). After 4 weeks of SF exposures, increases in VWAT macrophages (**h**), neutrophils (**i**), and M1 macrophages (**j**) emerged, and returned to within CTL values in SF 2R mice.

Mice exposed to SF exhibited increased food intake that began within a few days following the initiation of SF and was sustained throughout the duration of SF exposure (Fig. 1a). However, upon cessation of SF at 4 weeks, the increased food intake rapidly reverted back to baseline during the subsequent 2 weeks of recovery (Fig. 1a). As previously reported, no significant differences in body weight were present upon completion of the SF exposures at 4 weeks (27.2 ± 1.6 g in SF and 27.1 ± 1.5 g in CTL mice; p > 0.05)^2^,^4^. However, SF mice exhibited significantly increased visceral fat mass at 4 weeks of SF when compared to CTL. Furthermore, visceral fat mass returned to levels observed in controls 2 weeks after discontinuation of SF (Fig. 1b). Fasting HOMA values were higher in SF-exposed mice and returned to CTL values following 2 weeks of recovery (Table 1; Fig. 1c). *Ex vivo* assessment of adipocyte insulin sensitivity revealed reduced p-AKT/AKT ratios in VWAT adipocytes derived from SF 4w-exposed mice when compared to CTL mice, but such differences were abrogated in SF 2R adipocytes (Fig. 1d).

In conjunction with SF-induced increase in visceral white adipose tissue (VWAT), increased numbers of leukocytes, primarily consisting of neutrophils and macrophages, the latter particularly exhibiting M1 polarity, were identified after 4w SF, and nearly returned to CTL values in 2WR SF mice (Fig. 1e–i). Such changes were present across several VWAT depots (data not shown) but were particularly prominent in mesenteric VWAT (Fig. 1e–i). In parallel with such changes, 4w SF increased expression of *LBP* in VWAT adipocytes and macrophages (4.3 ± 0.6 and 5.8 ± 1.4 fold compared to CTL; n = 7; p < 0.0001), as well as increases in *IL-6* (3.7 ± 0.8 and 7.4 ± 2.6 fold; n = 7; p <  < 0.00010), and *NGAL* (4.4 ± 1.1 and 6.8 ± 2.0 fold; n = 6; p < 0.0001), with all three genes returning to CTL levels after 2-week recovery, except for IL-6 mRNA expression in macrophages which although decreased (1.9 ± 0.4 fold; n = 6; p < 0.001) in respect to SF 4w, remained elevated compared to CTL (p < 0.01). Similarly, plasma LBP, NGAL, and IL-6 protein levels were increased in 4w SF mice, and except for IL-6 returned to baseline levels (Table 1). A significant association between pAKT/AKT insulin slopes in mesenteric adipose tissues and corresponding LBP or NGAL plasma levels emerged (r^2^:0.47 and r^2^:0.56, respectively, p < 0.0001).

### Microbial community profiling

Regardless of recent debates^14^ fecal material is widely accepted as a proxy for intestinal microbiota studies^5^,^15^,^16^,^17^. Accordingly, fecal DNA was used for PCR reactions with universal primers^18^. PCR amplicons were sequenced, assembled in operating taxonomic units OTUs, taxonomically annotated, and used to assess the structure and membership of the gut microbial community. In total, 7,009,841 sequences were recovered and rarefied to 10,000 sequences/sample. Good’s coverage estimation^19^ showed near complete coverage of 0.99 ± 0.002, indicating that 99% of targets were sequenced at least twice. Sequences were assembled in 5995 OTUs which gains 232 ± 27.8 OTUs per sample. OTUs were attributed to 12 phyla, 26 classes, 44 orders, 74 families, and 117 genera (Supplementary materials 1s).

**Figure 2:**
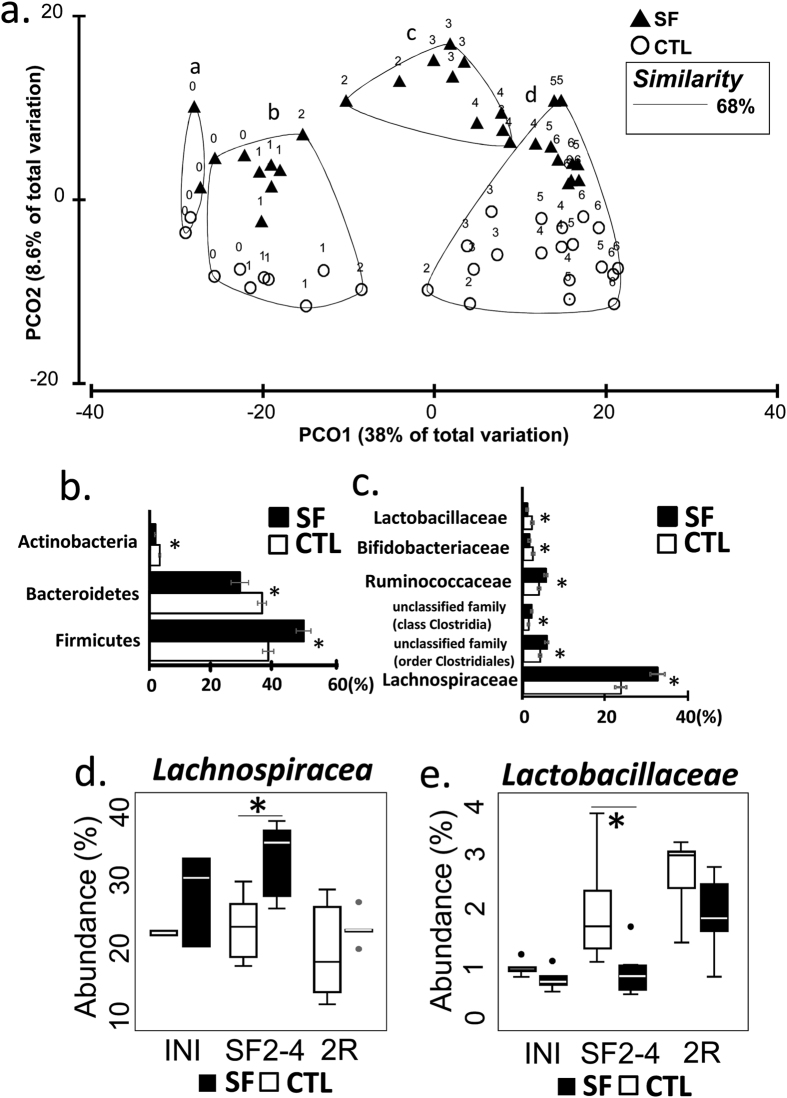
The properties of the gut microbial community are altered by SF treatment. (**a**) PCoA plot demonstrates structural changes in microbial communities over the period of 7 weeks, introduced by SF exposure followed by 2 weeks of sleep recovery. The major clusters (**a**–**d**) of individual points (averaged microbiota per treatment per day) were outlined by 68% similarity between data points (Bray-Curtis index), ANOSIM test confirms separation (p = 0.001). The data points are labelled by the week of experiment (0 week - a week of acclimation; 1–4 weeks - SF exposure, 5–6 week – recovery). (**b**) Differentially abundant bacterial phyla and (**c**) families in SF and CTL samples at weeks 2–4 (METASTATS, p < 0.02). (**d**,**e**) The abundance of *Lachnospiraceae* and *Lactobacillaceae* family members is different between SF and CTL groups at weeks 2–4 (W2–4) (*p < 0.002), but not at the initiation of treatment (INI) and at the end of recovery period (2R). Boxplots represent median and 25–75 percentiles.

To assess the effect of SF, the table of OTU abundances was used to calculate distances between samples using Bray-Curtis dissimilarity measure^20^. OTU abundances were averaged by treatment/day of collection, standardized, square root transformed and used to calculate dissimilarity matrix for Principal Coordinate analysis (PCoA). The Group-averaged clusters from Bray-Curtis similarities (“a”-“d”) were formed at 68% (arbitrary cut-off) similarity level and superimposed on PCoA plot, as described in Clarke and Warwick (2001)^21^. Superimposition demonstrated alteration of microbial community that occurred within 2 weeks of SF exposures (Fig. 2a). The clusters are significantly different from each other (ANOSIM, p < 0.001) and consist of samples: “a” - samples collected during early acclimation period, i.e., 7 and 5 days before SF treatment was initiated; “b” - CTL and SF samples spanning the period from late acclimation (3 and 1 days before SF treatment was initiated) and from 1–2 weeks of experiment; “c” - SF samples 2–4 weeks of treatment, and “d” - CTL samples weeks 3–6, one SF sample of day 28 (week 4) and SF-recovery samples week 5–6. However, while SF treatment altered community structure, the complexity of microbial populations remained unchanged between samples from cluster “c” and matching samples from clusters “b” and “d” (Simpson diversity index in the two-sided t-test, p = 0.29).

METASTATS comparison identified differentially abundant bacterial taxa in SF (samples from cluster “c”) and CTL groups (matching samples from clusters “b” and “d”). At the level of phylum, *Firmicutes* increased in abundance after SF treatment by 20%, while *Bacteroidetes* and *Actinobacteria*, were reduced by 20% and 50% abundance, respectively (METASTATS, p < 0.05) (Fig. 2b). At greater taxonomic resolution, SF exposure was associated with the expansion of *Lachnospiraceae and Ruminococcaceae* families, with addition of several lineages from *class Clostridia* and order *Clostridiales*. In contrast, during SF exposure families *Lactobacillaceae* and *Bifidobacteriaceae* were suppressed nearly 2-fold (Fig. 2c).

In an attempt to identify most prominent taxa affected by SF, we studied OTUs from cluster “b” (abundances >1%), and found 5 OTUs with abundances increased by >2-fold when compared to the CTL group (METASTATS, p < 0.02; Table 2). These OTUs were attributed to the order of *Clostridiales*, with 80% belonging to the family of *Lachnospiraceae*. The decrease of abundance >2-fold in response to SF (Table 2) is shown by other 5 OTUs, one of which was annotated as *Lactobacillaceae* genus. No differentially abundant OTUs from *Bifidobacteriaceae and Ruminococcaceae* families exhibited the same degree of alterations. This finding further justified our attention on the dynamics of *Lachnospiraceae and Lactobacillaceae* families (Fig. 2d,e). The abundance of *Lachnospiraceae* family increased after initiation of SF treatment (p < 0.002) and returned to control levels when the SF exposures were ceased (p = 0.44). In contrary *Lactobacillaceae* demonstrated opposite reaction the decrease of abundance during SF treatment (p = 0.0033) and return to CF level after 2 weeks of recovery (p = 0.17).

### Phenotype Arrays

**Figure 3:**
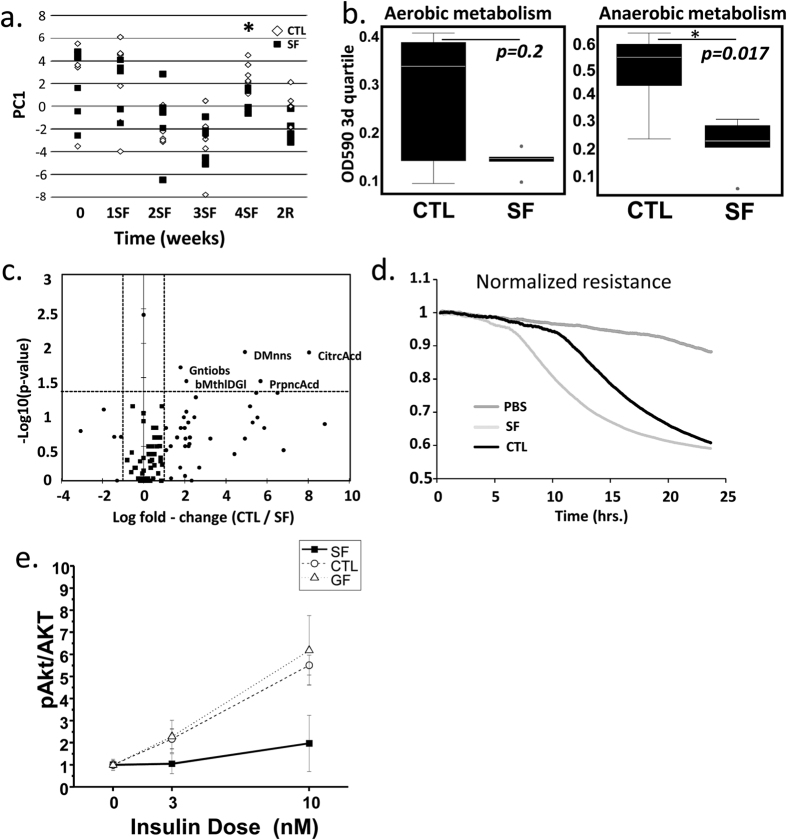
(**a**) The effect of SF on anaerobic substrate utilization increases separation of samples over the first principal coordinate (PC1) at 4-weeks of exposure, separation decreases after 2 weeks of recovery. (**b**) Boxplots compare the third quartiles of OD590 values across aerobic and anaerobic plates and shows no effect of 4w SF on aerobic microbial substrate utilization and inhibitory effect on anaerobic substrate utilization. (**c**) The volcano plot demonstrates the decrease of anaerobic utilization of 5 substrates in 4w SF microbial community. (**d**) Marked disruption of epithelial cell barrier occurred in monolayers of Human Normal Colon Cells (HNCC) exposed to SF-derived, but not to CTL-derived “fecal water” as demonstrated by mean TER changes. (**e**) VWAT insulin sensitivity is reduced in germ-free mice after conventionalization with SF-derived cecal microbiota as compared to CTL-derived or germ-free mice.

The basis for physiological profiling is the fact that structurally and functionally different microbial communities metabolize different sets of substrates^22^. To infer information about community dynamics, the rates of aerobic and anaerobic substrate utilization in CTL and SF microbial communities (n = 5/time point) were compared using fecal specimens collected before the start of SF treatment, at 1, 2, 3, and 4 weeks of SF and two weeks after cessation of SF treatment (2R). The results of PERMANOVA comparisons indicated SF did not affect that aerobic community metabolism. However, anaerobic microbial metabolism was significantly altered after 4 weeks of SF (PERMANOVA, p = 0.032), as illustrated by the increased separation of samples over the first principal coordinate (PC1) after 4-weeks of SF treatment. These differences were absent at 2R, indicating complete recovery of microbiota in the ability to metabolize available substrates (PERMANOVA, p = 0.84) (Fig. 3a). The comparisons of third quartiles of OD values across aerobic and anaerobic plates at 4 weeks confirmed that SF had no effect on aerobic, but suppressed anaerobic substrate utilization (Wilcoxon test, aerobic p = 0.2, anaerobic p = 0.017; Fig. 3b). Additional analysis identified significant changes at 4W SF in the metabolic rates associated with anaerobic utilization of 6 substrates (Fig. 3c). Microbial communities at 4W of SF decreased utilization of D-mannose and citric acid (both >400-fold reductions), gentiobiose, β-Methyl-D-glicoside and propionic acid (all reduced 3–4 fold). SF microbiota demonstrated increased anaerobic resistance to surfactant Niaproof 4 (p = 0.004), but CTL communities did not have such resistance (Supplementary Material 2s).

### “Fecal water” from stool samples after 5 weeks of SF exhibits enhanced colonic epithelium barrier disruption

*In vitro* experiments were performed to determine effects of gut microbiota on intestinal barrier. These effects may underlie SF-related increases in LBP levels. Trans-epithelial electric resistance (TER) measurements after treatment with PBS (vehicle control), samples of SF or CTL “fecal water”, a preparation of microbial-void water-soluble parts of fecal metabolome and proteome, were applied to confluent monolayers of both Human Normal Colon Cells (HNCC) and CACO-2 cells. Normalized resistance tracings in HNCC cell monolayers (Fig. 3d) showed rapid decreases in TER induced by contact with “fecal water” samples from SF, but not from CTL mice. Similar experiments with CACO-2 cells confirmed the immediate and sustained decreases in TER induced by SF “fecal water” (data not shown).

### Conventionalization of SF and CTL cecal microbiome in germ-free mice

Conventionalization experiments with microbiota from mice exposed to 4W of SF vs. CTL showed increased food intake (3.7 ± 0.7 vs. 3.2 ± 0.6 g/day/mouse; p = 0.05), but no differences in body weight gain or VWAT mass were apparent after 2 weeks. However, conventionalization with SF microbiota elicited markedly enhanced inflammatory responses in mesenteric and epididymal fat tissues when compared to either conventionalization with microbiota from CTL mice or mice that remained germ-free. These responses were characterized by increases in overall macrophage counts (CTL: 4.9 ± 2.7 vs. SF: 17.8 ± 6.7%% of live cells; p < 0.001; n = 8/group; non-conventionalized controls: 3.8 ± 2.1%; n = 4), neutrophils (CTL:16.6 ± 4.7% vs. SF: 49.2 ± 11.2% of CD11b^+^ cells; p < 0.001; n = 7/group; non-conventionalized controls: 11.7 ± 4.1%; n = 4), and a major shift toward M1 macrophage polarity as illustrated by the proportion of CD11c+ macrophages among CD11+ cells (CTL: 18.7 ± 5.1% vs. SF: 42.1 ± 10.2%; p < 0.001; n = 8/group; non-conventionalized controls:12.8 ± 3.1%; n = 4). Furthermore, increases in systemic HOMA-IR and systemic inflammatory markers (Table 3), as well as reduced pAKT/AKT insulin sensitivity in the epididymal fat of mice conventionalized with SF-derived microbiota emerged (Fig. 3e) thus recapitulating SF metabolic phenotype.

## Discussion

This study shows that long-term SF exposure (4 weeks) during the sleep period in mice leads to increased visceral fat mass, VWAT inflammation, as wells as systemic insulin resistance and increased leptin plasma levels. These effects are reversible upon cessation of SF exposure. Furthermore, increased numbers of M1 macrophages and neutrophils in VWAT, particularly in mesenteric adipose tissues, along with elevated IL-6, NGAL, LBP levels, were present in SF-exposed mice, suggesting a potential gut-derived transfer of microbially-produced substances to elicit an inflammatory response. Analyses of the gut microbiome further revealed that SF induces temporally coordinated changes in microbial communities and in their substrate processing properties, signifying a shift in microbial function despite a relatively minor, albeit significant effect on overall microbial community structure. Conventionalization of germ-free mice with microbiota from SF and CTL mice recapitulated the systemic and adipose tissue inflammatory responses and metabolic alterations induced by SF. Taken together, chronic perturbations in sleep continuity, mimicking highly prevalent human sleep disorders, elicit systemic and adipose tissue inflammation and disruption of metabolic homeostasis that, in part, mediated by SF-induced alterations in gut microbiota.

Some methodological considerations deserve a comment. First, the sleep fragmentation procedures used herein afford reproducibility, lack of measurable increases in stress hormones or circadian disruption, the absence of human contact, and preserved social settings, while allowing for *ad libitum* food and water access, all of which, could independently contribute to the gut microbiome alteration^2^. Furthermore, we have also shown that the current model of SF does not curtail sleep duration and that the episodic arousals in the context of preserved sleep duration primarily manifest as increased sleep propensity, thereby closely mimicking several human sleep disorders^23^. We should also point out that chronic SF induces an increase in tissue level of tumor necrosis factor α (TNF- α), and it is possible that the increased systemic levels of this cytokine may operate also as a potent pro-inflammatory agent, most likely via activation of the TNF p75 receptor^24^,^25^. Alternatively and more akin to our current line of thought, gut microbiome changes would represent the initial set of events, which would facilitate the occurrence of low-grade bacterial challenges from the gut, thereby prompting immune responses, as exemplified by increased LBP and NGAL plasma and adipose tissue expression levels, with the latter changes being particularly prominent in the mesenteric VWAT. Nevertheless, it remains unclear how SF in the host induces such changes in the gut microbiome. It is possible that SF may alter gastrointestinal tract motility or the gut milieu, or possibly act via vagally mediated effects^26^. Notably, children with obstructive sleep apnea, a highly prevalent condition associated with chronic SF, exhibit markedly elevated circulating levels of LBP, particularly when obesity is concurrently present^27^. Thirdly, we should stress that we opted not to implement administration of a high fat diet or to employ transgenic mice with heightened susceptibility to obesity, since we wished to examine the isolated effect of disrupted sleep on the gut microbiome in isolation, rather than potentially confound our findings with changes in microbial communities that derive from dietary or genetic manipulations^28^,^29^,^30^.

Current experiments illustrate not only the emergence of VWAT inflammation and insulin resistance as induced by chronically disrupted sleep, but also reveal the potential reversibility of such metabolic perturbation upon discontinuation of SF. However, it remains unclear whether such reversibility is circumscribed to the temporal window preceding the emergence of frank obesity^2^ or will still be operationally viable even at more advanced stages of SF duration, once that obesity has occurred and settled in. Notwithstanding, the current conventionalization experiments clearly indicate that SF-induced alterations in fecal microbiota promote increased colonic epithelial barrier disruption that leads to systemic and tissue inflammatory phenotypic changes and concurrent emergence of metabolic dysfunction. Indeed, evidence for infiltration of M1 polarized macrophages and neutrophils are early processes in the context of high fat diet-induced insulin resistance^31^, and most likely reflect the changes induced by SF on the microbiome-gut permeability axis. Furthermore, the induction of VWAT inflammatory changes coincided with the emergence of systemic and tissue insulin resistance, as illustrated by the increases in HOMA-IR and by the reduced pAKT/AKT responses to exogenous insulin in VWAT.

Modern studies have provided conclusive evidence that host obesity alters gut microbiota^8^,^32^ and that, in turn, gut microbiota mediate the occurrence of metabolic disorders^11^. The microbiocentric paradigm mechanistically connected consumption of a high-fat diet with metabolic disorders via development of metabolic endotoxemia (increased level of LPS in blood circulation)^29^,^33^. Recent work has indicated the synergistic effect of circadian perturbations on the high fat diet-microbiome metabolic deregulation^7^. In turn, our study demonstrated that SF experiments preserving the circadian light zeitgebers affect gut microbiota by disturbing community structure, as clusters “b” and “c” separate at day 9 (Fig. 2), and inducing the emergence of metabolic phenotype in conventionalization experiment. We demonstrated that SF treatment changes the ratio of *Firmicutes* to *Bacteroidetes* (FB-ratio) from 1 in CTL to 1.7 in SF. A similar trend was previously associated with the development of obesity^16^ and low-grade inflammation^34^. Even though the functional significance of FB ratio is disputable^35^,^36^,^37^ it sets an important indicator of structural modifications of gut microbiota. In our study, the expansion of *Firmicutes* was associated with an increase in abundance of *Lachnospiraceae* and *Ruminococcaceae* bacterial families. These highly fermenting bacteria are important for degradation of plant-derived fibers^38^, production of short chain fatty acids^7^,^39^, and, hence, the increase of energy harvest^40^,^41^,^42^,^43^. The growth of *Firmicutes* could be explained by the noted increase in food intake and accompanied by the changes in anaerobic substrate utilization. Indeed, all impacted bacterial families (*Lachnospiraceae*, *Ruminococcaceae*, *Lactobacillaceae*, and *Bifidobacteriaceae*) are comprised of anaerobes. The increased food intake cannot be the only single factor explaining the bacterial dynamics^26^ in our study, as SF microbiota had a tendency to converge to SC state (day 28) (Fig. 2a), while the food intake still remained elevated. The mechanisms underlying this phenomenon warrant future investigation in experiments involving paired-fed mice. Nevertheless, the functional properties of SF microbiota, remained abnormal, as demonstrated consequently by phenotype arrays, ECIS and the conventionalization experiments.

Unmistakably, the conventionalization with SF microbiota conveyed the features of metabolic phenotype to naïve recipients. However, the absence of body weight and VWAT mass gains in these experiments, although anticipated since such changes require more extended exposures to SF than 2 weeks^2^, lead to the assumption that the complete SF-metabolic phenotype is the result of more than a single pathway, the symptoms of obesity and inflammation deserving separate consideration. To offer a unifying hypothesis, we speculate that the SF-related excessive food intake leads to body weight gain and induces changes in gut microbiota; the latter, mediates the emergence of an inflammatory phenotype via mechanisms associated with low grade systemic endotoxemia. In turn, SF altered microbiota will transfer such inflammatory phenotype to germ-free recipients. Alternatively, as mentioned it is very likely that 2 weeks post-conventionalization constitute an insufficient period of time to achieve excessive weight gain, since weight gain during SF exposures usually requires 4–5 weeks^2^.

The altered gut community (elevated *Lachnospiraceae* and decreased *Lactobacteriaceae*) was complemented by elevated IL-6, NGAL, and LBP levels indicating induction of inflammatory processes perhaps due to the leakage of microbial products into circulation. In earlier studies, the pro-inflammatory effect of innate Gram-positive bacteria (*Firmicutes*) was demonstrated in a mouse model of dextran sodium sulfate (DSS)-induced colitis^44^. The study denoted a prominent contribution of bacteria from the flagellated *Lachnospiraceae* family in the development of intestinal inflammation^44^,^45^. The bacterial flagellin, recognized by TLR5 at the basolateral sites of colonic epithelial cells, induces CCL2 secretion and recruitment of inflammatory macrophages^46^. The contribution of *Lachnospiraceae* to the onset of metabolic dysfunction was additionally confirmed in colonization experiments with germ-free ob/ob mice^47^. In germ-free recipients, colonization with *Lachnospiraceae* strain AJ110941 recapitulated the metabolic phenotype and caused the increase in fasting blood glucose, increase of liver and mesenteric adipose tissue weight, decreased plasma insulin levels and HOMA-β values, leading to speculation that *Lachnospiraceae* facilitate translocation of LPS from the alimentary tract into the blood^47^. This speculation is further supported by the observed decline of barrier protective bacteria (*Lactobacteriaceae* family^29^,^48^,^49^) and by our *in vitro* ECIS experiments, that confirms the barrier disruptive properties of fecal water prepared from feces of SF exposed mice. Thus, the discovered increase in relative abundance of *Lachnospiraceae* and loss of *Lactobacteriaceae* during the development of SF-induced metabolic phenotype may have important therapeutic implications, seeing that high fat diet-induced metabolic phenotype favorably responds to interventions with antibiotics^29^, pre-biotics^50^ and pro-biotics^48^,^49^.

In summary, chronic sleep disruption leads to increases in fat mass, induces selective alterations in gut microbiota that elicit increased gut permeability and concurrent systemic and adipose tissue inflammatory changes accompanied by insulin resistance, in both conventional and conventionalized mice. These findings open the way for future interventional approaches aimed at restoration of gut microbiota to prevent or palliate the multiple end-organ morbidities associated with chronic SF.

## Methods

### Animals

Adult male C57BL/6J mice from Jackson Laboratories (8-week old, ~22–23 grams; Bar Harbor, ME, USA), were housed in groups of five for 2 weeks. Mice were fed normal chow diet and water *ad libitum* and maintained in a 12-h light/dark cycle (light on 7:00 am to 7:00 pm) at a constant temperature (24 ± 1 °C). Mice were randomly assigned to sleep fragmentation (SF, n = 30) exposures or sleep control (CTL, n = 30) conditions. Stool was collected every other day for 42 days in a subset of animals (SC (n = 6), SF (n = 7), and at day 28 and 42 the entire available population was sampled. Animal experiments were performed according to protocols approved by the IACUC of the University of Chicago (72043, 72078).

### Sleep Fragmentation Paradigm

The sleep fragmentation was induced as previously described^23^. Briefly, it employs intermittent tactile stimulation using a near-silent motorized horizontal bar sweeping just above the cage floor from one side to the other. Since on average 30 episodes of arousal per hour occur in patients with severe OSA (i.e., every 2 min), our aim was to mimic closely the more severe disease condition, and thus, a 2-min interval between each sweep was implemented during the light period (7:00 am to 7:00 pm). SF was implemented for a period of 4 weeks (SF 4w) and then discontinued in a subset of mice (n = 5) for a recovery period of 2 weeks (SF 2R).

### Food consumption and body weight

Food consumption per cage was recorded daily and body weight was measured every other day at the middle of the light period. Food consumption was calculated by dividing the daily cage chow utilization by the number of mice in the cage. Body weight gain was determined by subtracting the body weight on the first day of SF exposure from the body weight on subsequent days.

### Glucose tolerance test (IGTT)

IGTT was performed after 4-week of exposure (SF 4w), following the 2-week recovery period (SF 2R), and in CTL. Mice were injected with glucose (2 mg/g body weight, i.p.) after 3 hours of fasting. Water was available during the fasting period. Blood glucose was measured using an OneTouch Ultra 2 glucometer (Life Scan; Milpitas, CA). Blood samples were collected from the tail vein at 0, 4, 15, 30, 60, 90 and 120 min. Glucose response during IGTT was evaluated by estimating the total area under the glycemia index *vs*. time curves.

### Insulin tolerance test (ITT)

Mice were injected intraperitoneally with humulin (0.25 U/kg of body weight) after 3 hours of fasting in both SF 4w, SF 2R, and CTL mice. Blood was collected via the tail vein from each mouse, and blood glucose was measured using an OneTouch Ultra2 glucometer (Life Scan; Milpitas, CA). Blood samples for insulin determination were obtained from the cut tip of the tail at 0, 4, 15, 30, 60 and 120 min following injection. Insulin resistance was assessed using the homeostasis model assessment (HOMA-IR) equation (fasting insulin × fasting glucose/22.5). ITT glycemic trajectories were also analyzed for differences in insulin sensitivity as previously described^51^. Mice undergoing ITT were not assessed for IGTT and *vice-versa*.

### Insulin Sensitivity Assay

Adipocyte insulin sensitivity was assessed in adipocytes, derived from visceral fat tissue, using Western blotting for phosphorylated and total Akt (antiphospho-Akt [Ser473] and anti-Akt; Cell Signaling Technology, Danvers, MA) as previously described^52^.

### Isolation of stromal-vascular fraction (SVF) and flow cytometry analysis

Isolation and analysis were performed as described previously^3^,^4^. Adipose tissue macrophages were defined as CD11b+ cells, from which M1 and M2 macrophages were identified as CD11c+ or CD206+ cells, respectively. Neutrophils identified as CD11b+ and Ly6Chigh+ cells^53^. All antibodies were from Biolegend (San Diego, CA).

### Leptin, lipopolysaccharide-binding protein (LBP), neutrophil gelatinase-associated lipocalin (NGAL) and IL-6 expression and plasma levels

Plasma leptin and IL-6 levels were measured using commercial kits (Millipore; St. Charles, Missouri, USA and Biolegend, San Diego, CA, USA, respectively) as described by the manufactures. NGAL is a 25-kDa secretory glycoprotein that belongs to the lipocalin family and is expressed in many tissues and cells in addition to adipose tissue, including leucocytes (macrophages and neutrophils). NGAL expression is induced by various pro-inflammatory stimuli through Nuclear factor-kB activation, and plasma levels reflect not only inflammatory status elicited by LPS or other bacterial products but are also associated with insulin resistance^54^. Similarly, lipopolysaccharide-binding protein (LBP) is released by various cells including macrophages and adipocytes and is associated with insulin resistance^55^. LBP and NGAL assays were performed using commercial ELISA assays (Cell Sciences, Canton, MA, USA, and R&D Systems, Minneapolis, MN) according to the manufacturer’s instructions. Total RNA was isolated from VWAT adipocytes and macrophages using automated RNA extraction (Promega, Madison, WI) and quantified on a Nanodrop 2000 (Ambion, Austin, TX, USA). Expression of *LBP* and NGAL was examined using RT-PCR using specific TaqMan primers. Quantitative RT-PCR (qRT-PCR) was performed for mRNAs in triplicate in ABI PRISM 7500 System (Applied Biosystems, Foster City, CA, USA). Ribosomal 18S rRNA was used as a reference gene to normalize the expression ratios. The mean cycle number (Ct) values of the 18S Ct and the gene of interest Ct were calculated. The relative expression of the gene of interest was calculated using the 2-ΔΔCt method.

### 16S rRNA gene tags analysis

Fecal DNA was isolated using Stool Fast Mini Kit (Qiagen). The 16S rRNA tag libraries were generated using the set of indexed primers (V4 hypervariable region) and sequenced on Illumina MiSeq platform^18^. The unidirectional reverse sequences (mean length 150 bp) were collected, processed and annotated vs. RDP database (v.9)^56^ using Mothur software^57^ as described^58^,^59^. Metastats software^60^ was used to determine differentially abundant taxa; PCoA and ANOSIM tests (Primers 6)^61^ were used to compare groups and time points.

### Phenotype Arrays

Metabolic arrays (Gen3 MicroPlates (BIOLOG Inc., CA)) were used to determine sets of substrates metabolized by microbial communities of CTL and SF treated animals. One freshly collected fecal pellet was dissolved in 1 ml of inoculation fluid IFa^7^. The homogenate was centrifuged briefly to remove debris; supernatant (15 μl) was used to initiate aerobic and anaerobic growth in MicroPlates. Anaerobic plates were prepared, inoculated and incubated in an anaerobic chamber (Coy, Grass Lake, MI) at 37 °C, while aerobic plates were handled and incubated at room air. Optical density (OD 590 nm) was measured at the end of 18 hours incubation. The OD values for 96 substrates were log10 transformed and normalized separately across anaerobic and aerobic plates. The Euclidian distances matrix between samples was calculated and used in PERMANOVA test to estimate the significance of SF effect. Nonparametric Wilcoxon test was used to determine differentially metabolized substrates.

### Conventionalization Experiments

The freshly excised ceca of SF and CTL mice were prepared as previously described^8^ and functionally active microbiota was transferred into germ-free (GF) recipient mice by oral gavage (200 μl cecal content in the sterile saline buffer). GF mice conventionalized with SF or CTL microbiota were euthanized at 2 weeks post-colonization. Their plasma and VWAT were processed as described above.

### Electrical cell impedance (ECIS)

The “fecal water”, a preparation of water-soluble parts of fecal metabolome and proteome, was used to determine barrier disruptive properties of gut microbiota that may underlie endotoxemia. Fresh fecal samples from C57BL/6J CTRL mice and mice exposed to SF for 5 weeks were pooled by cage and mixed in PBS to final concentration of 1 mg/ml (w/v), cleared by centrifugation (1 min, 5,000 rpm), filtered using 20 μm membrane filter (Millipore), and applied (15 μl per well; 10% (v/v)) to ECIS system (Applied Biophysics, Troy, NY) to determine change of electrical resistance across monolayers of CACO-2 (ATCC, Manassas VA) and Normal Human Colon Cells (NHCC) (Applied Biological Materials Inc. Canada).

### Statistical analysis

All values are expressed as mean ± standard deviation (SD). Analyses of variance procedures followed by *post-hoc* tests and Student *t* tests were used to compare the results between SF 4w, SF 2R, and CTL groups. In all cases, a two-tailed p-value of <0.05 was considered to achieve statistical significance.

## Additional Information

**How to cite this article**: Poroyko, V. A. *et al*. Chronic Sleep Disruption Alters Gut Microbiota, Induces Systemic and Adipose Tissue Inflammation and Insulin Resistance in Mice. *Sci. Rep.* **6**, 35405; doi: 10.1038/srep35405 (2016).

## Supplementary Material

Supplementary Dataset 1

Supplementary Dataset 2

## Figures and Tables

**Table 1:** Systemic insulin resistance, leptin and inflammatory markers following SF and recovery.

	SF 4w (n = 12)	SF 2R (n = 12)	CTL (n = 12)
HOMA-IR	8.7 ± 2.4^§^	3.9 ± 0.9	3.6 ± 0.6
Leptin (ng/ml)	0.89 ± 0.42^§^	0.73 ± 0.32	0.78 ± 0.28
NGAL (ng/ml)	146.3 ± 39.4^§^	68.9 ± 11.4	59.4 ± 9.6
LBP (ng/ml)	58.9 ± 14.2^§^	23.6 ± 6.8	21.4 ± 6.5
IL-6 (pg/ml)	9.2 ± 3.4^§^	7.5 ± 2.9^¥^	3.6 ± 0.8

^§^SF 4w vs. SF 2R or CTL – p < 0.01; ^¥^SF 2R vs. CTL – p < 0.05.

**Table 2:** Significantly different OTUs identified in CTL and SF groups.

OUT*	CTL	SF	CTL to SF fold change	p-value	Taxonomic attribution
Otu00025	0.013 ± 0.003**	0.003 ± 0.001	↓4.3	0.007	*Lactobacillaceae genus*
Otu00036	0.007 ± 0.015	0.015 ± 0.002	↑2.14	0.002	*Lachnospiraceae*,*unclassified genus*
Otu00028	0.005 ± 0.016	0.016 ± 0.003	↑3.2	0.025	*Lachnospiraceae*,*unclassified genus*
Otu00037	0.005 ± 0.012	0.012 ± 0.002	↑2.4	0.001	*Lachnospiraceae*,*unclassified genus*
Otu00011	0.004 ± 0.029	0.029 ± 0.008	↑7.25	0.005	*Lachnospiraceae*,*unclassified genus*

^*^OTUs exceeding 2 folds of change and annotated to the genus level are listed.

^**^Median relative abundance ± standard error.

**Table 3:** Systemic insulin resistance and inflammatory markers following conventionalization of germ-free mice by SF and CTL fecal microbiome.

	TF-SF (n = 8)	TF-CTL (n = 8)	Non-TF Germ-Free (n = 4)	P-value (TF-SF vs. TF-CTL or Non-TF)
HOMA-IR	6.2 ± 2.5	3.4 ± 1.4	3.4 ± 1.5	<0.01
NGAL (ng/ml)	198.4 ± 46.8	77.6 ± 35.7	64.3 ± 45.1	<0.01
LBP (ng/ml)	112.6 ± 23.8	44.9 ± 22.1	27.9 ± 20.2	<0.01
IL-6 (pg/ml)	35.4 ± 8.4	19.5 ± 7.2	12.6 ± 5.5	<0.05
